# A five‐mRNA signature associated with post‐translational modifications can better predict recurrence and survival in cervical cancer

**DOI:** 10.1111/jcmm.15270

**Published:** 2020-04-19

**Authors:** Mingyi Ju, Aoshuang Qi, Jia Bi, Lan Zhao, Longyang Jiang, Qiang Zhang, Qian Wei, Qiutong Guan, Xueping Li, Lin Wang, Minjie Wei, Lin Zhao

**Affiliations:** ^1^ Department of Pharmacology School of Pharmacy China Medical University Shenyang City Liaoning China; ^2^ Liaoning Key Laboratory of Molecular Targeted Anti‐Tumor Drug Development and Evaluation China Medical University Shenyang City Liaoning China

**Keywords:** biomarkers, cervical cancer, post‐translational modifications, activated CD4 memory T cell, tumour‐infiltrating immune cells

## Abstract

High mortality of patients with cervical cancer (CC) stresses the imperative of prognostic biomarkers for CC patients. Additionally, the vital status of post‐translational modifications (PTMs) in the progression of cancers has been reported by numerous researches. Therefore, the purpose of this research was to dig a prognostic signature correlated with PTMs for CC. We built a five‐mRNA (GALNTL6, ARSE, DPAGT1, GANAB and FURIN) prognostic signature associated with PTMs to predict both disease‐free survival (DFS) (hazard ratio [HR] = 3.967, 95% CI = 1.985‐7.927; *P* < .001) and overall survival (HR = 2.092, 95% CI = 1.138‐3.847; *P* = .018) for CC using data from The Cancer Genome Atlas database. Then, the robustness of the signature was validated using GSE44001 and the Human Protein Atlas (HPA) database. CIBERSORT algorithm analysis displayed that activated CD4 memory T cell was also an independent indicator for DFS (HR = 0.426, 95% CI = 0.186‐0.978; *P* = .044) which could add additional prognostic value to the signature. Collectively, the PTM‐related signature and activated CD4 memory T cell can provide new avenues for the prognostic predication of CC. These findings give further insights into effective treatment strategies for CC, providing opportunities for further experimental and clinical validations.

## 1 INTRODUCTION

Cervical cancer (CC) is the fourth most common gynaecological malignancies among women worldwide and represents a major global health challenge.^1^ There were estimated 569 847 new CC cases and 311 365 deaths worldwide in 2018. CC is the leading cause of cancer death in 42 countries among women.^1^ Despite the phenomena that there are plentiful methods of treatment for CC, a considerable number of patients succumb to CC metastasis and recurrence. So, it would be a wonderful landscape if metastasis and recurrence of CC could be predicted by some biomarkers. Therefore, identifying novel prognostic biomarkers and potential therapeutic targets is necessary to improve the survival of CC patients.

Post‐translational modifications (PTMs), such as phosphorylation, glycosylation and ubiquitination, play essential roles in life. PTMs, such as glycoproteins and sumoylation, usually act as key players during metastasis and angiogenesis.^2^, ^3^ When some genes, such as p53 which is associated with cancer, modified by PTMs, tumorigenesis could usually be accelerated in many cancers.^4^ Subsequent research illustrated that mutation of acetylation and ubiquitination PTM sites had great importance in cancer.^5^ Given above, we recognized that PTMs acted as essential roles during tumorigenesis and metastasis.

Previous study has discovered many prognostic biomarkers associated with PTMs for cancers, such as hepatocellular carcinoma,^6^ colorectal cancer^7^ and breast cancer.^8^ However, limited studies have systematically investigated the PTMs and its prognostic value in CC patients. To date, many clinical researches have proved that the therapeutic strategies that target the PTM pathway for cancer therapy were safe and well‐tolerated.^9^, ^10^ Therefore, in this study, we were dedicated to mining a gene signature related to PTMs in CC.

In this study, we developed and validated a five‐mRNA (GALNTL6, ARSE, DPAGT1, GANAB and FURIN) prognostic signature for CC from PTM‐related gene set in The Cancer Genome Atlas (TCGA) training set. Using GSE44001 testing set, we proved stability and reliability of the signature. Functional annotation of the downstream target genes of the genes in the signature revealed that these downstream target genes were associated with cell‐signalling networks and immunity in which PTMs usually acted as a vital role. Given this, we further quantified the cellular composition of the immune response in CC in order to investigate its relationship with the PTM‐related prognostic signature and survival by using the CIBERSORT algorithm. The results showed that activated CD4 memory T cell was also an independent factor for disease‐free survival (DFS), with lower AUC value than our five‐mRNA prognostic signature, and could add additional prognostic value to our PTM‐related signature. These findings provide further insights into effective treatment strategies for CC, providing opportunities for further experimental and clinical validations. Moreover, detailed analysis of the cellular immune response in CC has the potential to enhance clinical prediction and to identify candidates for immunotherapy.

## 2 METHODS

### 2.1 Patient data

Gene expression data and associated clinical characteristics of CC patients were downloaded from The Cancer Genome Atlas (TCGA, http://cancergenome.nih.gov/). This cohort has 304 CC patients with the corresponding gene expression data and clinical information. Then, 291 patients were included after 13 patients were removed due to overall survival (OS) time is zero. Out of them, 272 with complete follow‐up information, including OS status and time and disease‐free status and time, were included in our training data set. The general clinical characteristics are listed in Table 1.

**TABLE 1:** Clinical pathological parameters of patients with cervical cancer in this study

Clinical characteristics	TCGA (N = 272)		
	n	%	Dead number
Age (y)			
≤46	138	50.74	29
>46	134	49.26	36
Ethnicity			
Not Hispanic or Latino	151	87.79	27
Hispanic or Latino	21	12.21	3
FIGO stage			
I + II	210	78.95	43
III + IV	56	21.05	22
Pathologic T			
T1 + T2	198	89.19	36
T3 + T4	24	10.81	12
Pathologic N			
N0	124	70.06	15
N1	53	29.94	17
Pathologic M			
M0	104	92.04	17
M1	9	7.96	4
Tumour events			
No	224	82.35	33
Yes	48	17.65	32
Birth control pill history			
No	83	55.70	19
Yes	66	44.30	11
Radiation therapy			
No	46	31.29	10
Yes	101	68.71	31
Tobacco smoking history			
≤1	136	56.43	28
>1	105	43.57	31
Pregnancy history			
No	16	6.61	4
Yes	226	93.39	55

Abbreviation: FIGO, International Federation of Gynecology and Obstetrics; TCGA, The Cancer Genome Atlas.

Microarray data sets including gene expression profiles and corresponding clinical information data of GSE44001 were downloaded from the Gene Expression Omnibus database (GEO, https://www.ncbi.nlm.nih.gov/geo/). GSE44001 was conducted by GPL14951 (Illumina HumanHT‐12 WG‐DASL V4.0 R2 Expression BeadChip), including 300 CC samples which were enrolled in our testing data set. Among them, 262 participants had recurrence or metastasis and 38 participants did not.

### 2.2 Construction and confirmation of a prognostic signature

**FIGURE 1:**
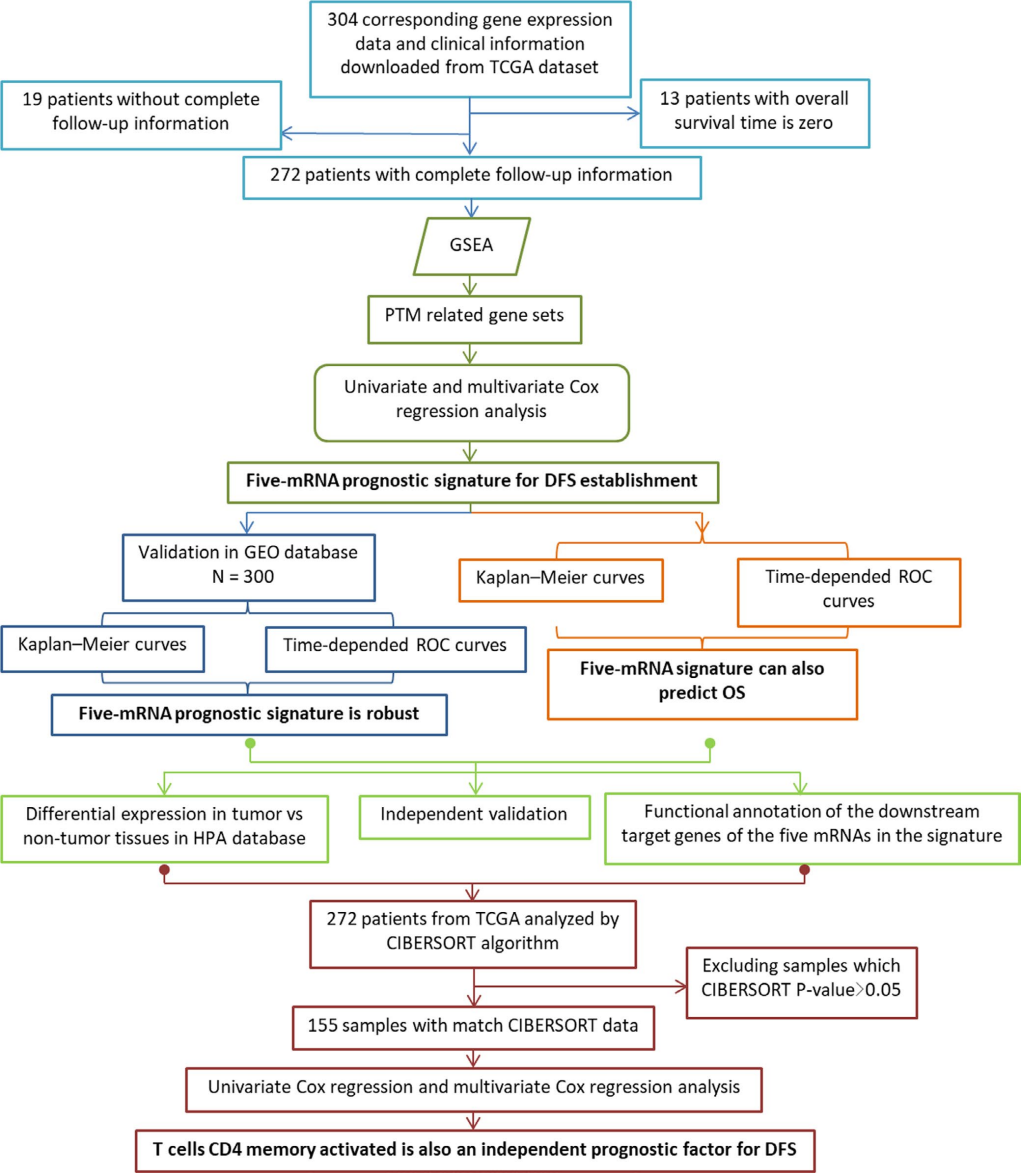
Overview of the analytic pipeline of this study

The overall design and flow diagram of this study are presented in Figure 1. For the reason of significant impact of PTMs on tumour progression, Gene Set Enrichment Analysis (GSEA) (http://www.broadinstitute.org/gsea/index.jsp) was performed to explore genes enriched in the PTM pathway which showed significant differences between patients with recurrence or metastasis and patients without. Subsequently, we analysed these genes by using Cox regression analysis. Thus, a prognostic signature was constructed for CC.

Next, a risk score was calculated for each patient from training and testing sets according to expression levels of the genes (expi) and coefficients of multivariate Cox regression analysis (bi). Then, patients were divided into high‐risk and low‐risk groups in either cohort by median risk score values, respectively. The formula used was as follows:

$$\text{Risk score}=\sum_{i=1}^{n}\exp i*bi$$

Subsequently, receiver operating characteristic (ROC) curves were plotted based on the risk scores and the survival status of each patient to compare the predictive accuracy of the gene signature. Kaplan‐Meier (K‐M) curves were carried out to compare survival and recurrence or metastasis risk between high‐risk and low‐risk groups. P values from log‐rank tests were calculated, and P value less than 0.05 was considered statistically significant.

Next, the Human Protein Atlas (HPA) database (http://www.proteinatlas.org/) was used to validate the expression of the genes on translational level in CC tissue compared with normal tissue. Gene Expression Profiling Interactive Analysis 2 (GEPIA 2) (http://gepia2.cancer‐pku.cn/#index) was used to explore the relationship between the expression of the genes and survival of CC.

### 2.3 Differentially expressed genes screening in TCGA data set of CC

We firstly normalized the gene expression profiles of TCGA by log2 transformation. Thus, 32 323 mRNAs were enrolled in our training set for further analysis. Then, we used the coding language R software to analyse the differentially expressed genes (DEGs). The DEGs were obtained with |log2 fold change| > 1 and adjusted *P* value < .05 as selection criteria for subsequent analysis. 1,190 mRNAs met our criteria from a total of 60 483 mRNAs were screened out as DEGs, including 613 up‐regulated mRNAs and 577 down‐regulated mRNAs.

### 2.4 Protein‐protein interaction network of DEGs in CC

In order to excavate the hub genes within the gene regulatory networks, protein‐protein interaction (PPI) network of DEGs was constructed by Search Tool for the Retrieval of Interacting Genes (STRING) Database (https://string‐db.org) and subsequently was visualized by Cytoscape software.

### 2.5 Functional and pathway enrichment analysis

For a more in‐depth investigation of the functions of the downstream target genes of the five genes, the Database for Annotation, Visualization and Integrated Discovery (DAVID) database (https://david.ncifcrf.gov/) was used to enrich biological themes on GO terms and on KEGG pathway maps. *P* value < .05 was set as the cut‐off criterion.

### 2.6 Evaluation of tumour‐infiltrating immune cells

Normalized gene expression data were used to infer the relative proportions of 22 types of infiltrating immune cells using the CIBERSORT algorithm.^11^ Gene expression data sets were prepared using standard annotation files and data uploaded to the CIBERSORT web portal (https://cibersort.stanford.edu), with the algorithm run using the default signature matrix at 1000 permutations. Here, we applied the original CIBERSORT gene signature file LM22 which defines 22 immune cell subtypes and analysed data sets of CC from TCGA grouped by disease‐free status. Subsequently, we selected 155 samples (consisted of 128 with recurrence or metastasis and 27 without recurrence or metastasis) which met the requirements of CIBERSORT *P* value < .05.

### 2.7 Statistical analysis

The expression profiles of mRNAs from TCGA and GEO were shown as raw data, and each mRNA was normalized by log2 transformation for further analysis. Statistical analysis was performed by using GraphPad Prism version 7.0 or SPSS version 19.0 software package. A two‐tailed *P* < .05 was considered statistically significant.

## 3 RESULT

### 3.1 Identification of a PTM‐related mRNA signature which can predict both DFS and OS of CC

**FIGURE 2:**
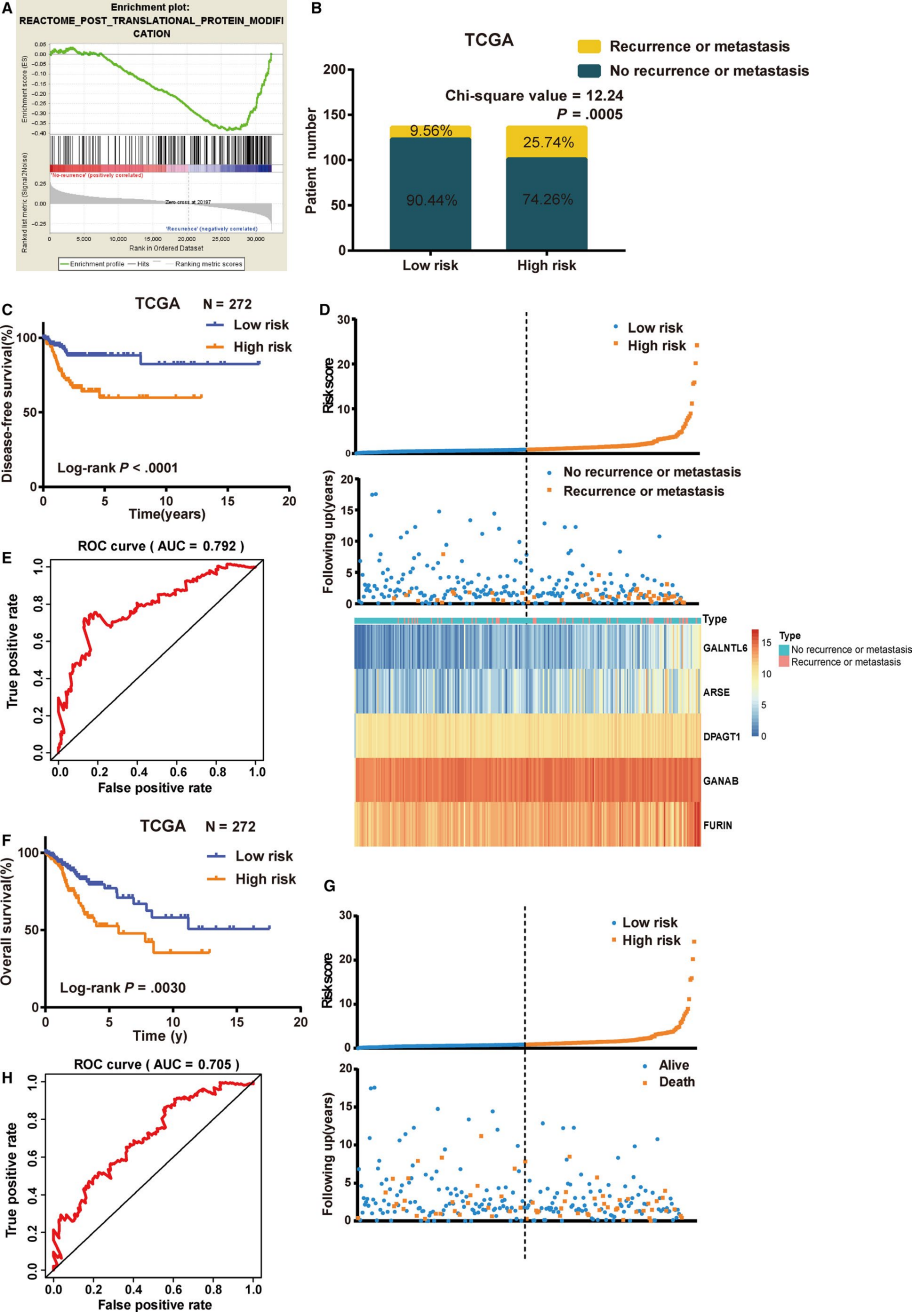
Construction of the five‐mRNA prognostic signature for DFS and OS of CC in the training set. A, Enrichment plots of gene set which were significantly differentiated between recurrence or metastasis and no recurrence or metastasis tissues in post‐translational protein modification pathway by using GSEA. B, Recurrence or metastasis rate in low‐ and high‐risk score groups. C, K‐M curves of DFS of low‐ and high‐risk groups. D, The distribution of risk scores, patient disease‐free status and gene expression levels. E, ROC curve for the 5‐y DFS prediction by the five‐mRNA signature. F, K‐M curves of OS of low‐ and high‐risk groups. G, The distribution of risk scores and patient OS status. H, ROC curve for the 5‐year OS prediction by the five‐mRNA signature. The black dotted line represents the median risk score cut‐off dividing patients into low‐risk and high‐risk groups. CC, cervical cancer; DFS, disease‐free survival; GSEA, Gene Set Enrichment Analysis; OS, overall survival

For the sake of the vital status of PTMs in the progression of cancers,^12^, ^13^, ^14^ GSEA was employed to identify the gene sets enriched in the pathway of PTMs by using mRNA expression files grouped by disease‐free status from the training set (Figure 2A). Thus, 71 genes, displayed yes in column of core enrichment, were obtained for univariate Cox regression analysis (Table S1). Then, we got 18 mRNAs with normalized *P* values < .05 which were correlated with the DFS of patients with CC (Table S1). Finally, as a result of multivariate Cox regression analysis, a five‐mRNA (GALNTL6, ARSE, DPAGT1, GANAB and FURIN) prognostic model was developed to predict prognosis using a risk score method. Hence, the CC risk score system was built as follows: risk score= (1.31878 × expression value of GALNTL6) + (1.09035 × expression value of ARSE) + (1.45048 × expression value of DPAGT1) + (1.96913 × expression value of GANAB) + (1.38201 × expression value of FURIN).

According to this formula, each patient was endowed a risk score. Then, 272 patients in the training set were classified into high‐risk and low‐risk groups by the cut‐off value of 0.902 as the median value of risk score. Chi‐square analysis showed that patients with recurrence or metastasis had a higher likelihood of being high‐risk patients than those without (*P* = .0005, Figure 2B), implying that higher risk score was associated with tumour recurrence or metastasis. K‐M curves showed that patients in high‐risk groups tended to have poorer clinical outcomes compared with those in low‐risk groups (log‐rank *P* < .0001; Figure 2C). The distribution of gene risk scores, patients’ disease‐free status and gene expression levels of 272 CC patients are shown in Figure 2D. Furthermore, AUC value of 5‐year DFS was 0.792, which illustrated the great accuracy of the combined five‐mRNA signature in DFS prediction (Figure 2E).

In view of predictive function of the signature of DFS, we curious that whether the signature can also predict OS. Surprisingly, K‐M curves illustrated that patients in high‐risk groups had significantly shorter OS when compared with those in low‐risk groups (log‐rank *P* = .0030; Figure 2F). The distribution of gene risk scores and patients’ OS status in CC samples showed in Figure 2G. Patients with high‐risk scores showed poor clinical outcomes compared with those with low‐risk scores. In addition, AUC value of 5‐year OS was 0.705, implying a high OS prediction performance (Figure 2H).

### 3.2 The five‐mRNA prognostic signature is robust in CC patients

**FIGURE 3:**
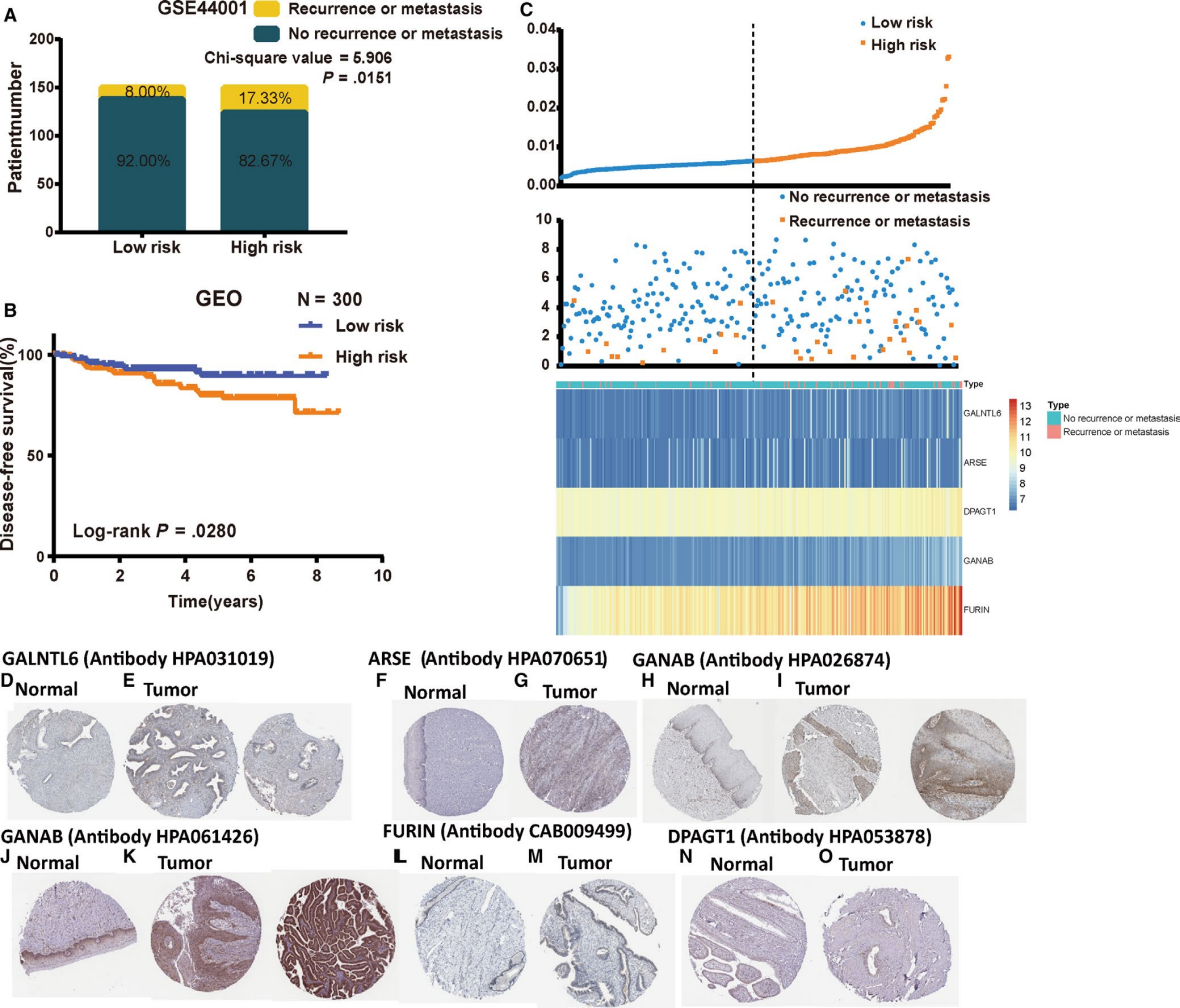
Validation of the five‐mRNA prognostic signature in the testing set and the HPA database. A, Recurrence or metastasis rate in low‐ and high‐risk score groups. B, K‐M curves of DFS of low‐ and high‐risk groups. C, The distribution of risk scores, patient disease‐free status and gene expression levels. D, GALNTL6 expression was negative in 2 normal samples (antibody HPA031019). E, GALNTL6 expression was medium in 1 of 12 CC samples (left) and low in 3 of 12 samples (right) (antibody HPA031019). F, ARSE expression was negative in 3 normal samples (antibody HPA070651). G, ARSE expression was low in 2 of 10 CC samples. H, GANAB presented low expression in normal tissue from 3 samples. I, GANAB presented medium (left) or even (right) high expression in 12 CC samples (antibody HPA026874). J, GANAB presented medium expression in normal tissue from 3 samples. K, GANAB presented medium (left) or even high (right) expression in 9 of 10 CC samples (antibody HPA061426). L, FURIN expression was negative in 3 normal samples. M, FURIN expression was low in 2 of 11 CC samples (antibody CAB009499). DPAGT1 presented medium expression level both in normal (N) and CC tissue (O) (antibody HPA053878). CC, cervical cancer; DFS, disease‐free survival; HPA, Human Protein Atlas

To validate predictive ability of the five‐mRNA signature, we applied the signature to the testing set (GSE44001). In testing set, we used the same risk score model to calculate each patient's risk score associated with DFS in testing set. Then, 300 patients were divided into high‐ and low‐risk groups using the median of the risk scores value of 0.0064. Chi‐square analysis showed that patients with recurrence or metastasis had a higher likelihood of being high‐risk patients than those without (*P* = .0151; Figure 3A), implying that higher risk score was associated with tumour recurrence or metastasis which was consistent with our previous findings. K‐M curves showed great utility in predicting DFS with *P* value of .028 (Figure 3B). In addition, the distribution of gene risk scores, patients’ disease‐free status and gene expression levels of 300 CC patients are shown in Figure 3C.

### 3.3 The expression of most of the five mRNAs is higher in CC tissue compared with normal tissue in HPA database

According to the immunohistochemical analyses in HPA database, the moderate or high staining intensity of these four proteins (GALNTL6, ARSE, GANAB and FURIN) in CC tissues contrasted sharply with the low intensity or lack of staining in normal tissues (Figure 3D‐M), while DPAGT1 (Figure 3N,O) did not show a significant difference. Given this, we can make a conclusion that the expression of most of the five mRNAs is significantly different between CC tissues and normal tissues, and usually higher in CC.

### 3.4 The five‐mRNA signature is an independently prognostic factor in CC patients

**FIGURE 4:**
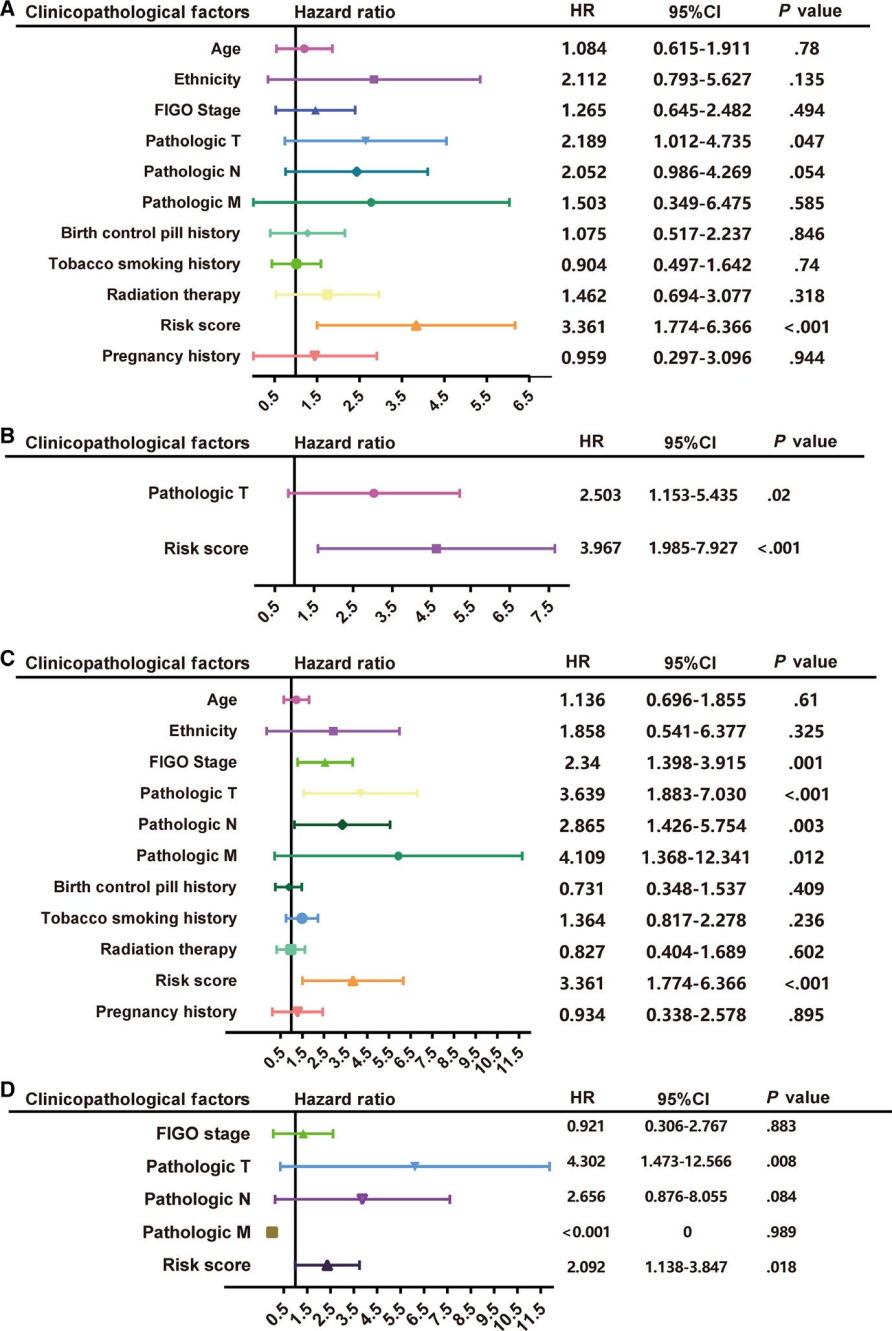
The five‐mRNA signature is an independent prognostic factor in CC patients. A, Univariate Cox regression of prognosis factor for DFS. B, Multivariate Cox regression of prognosis factor for DFS. C, Univariate Cox regression of prognosis factor for OS. D, Multivariate Cox regression of prognosis factor for OS. Unadjusted HRs (boxes) and 95% confidence intervals (horizontal lines) are showed in the figure. Box size is inversely proportional to the width of the confidence interval. CC, cervical cancer; DFS, disease‐free survival. HR, hazard ratio; OS, overall survival

In order to investigate the independence of the prognostic signature, we used Cox regression for further analysis in training set, combined with clinicopathological factors. Firstly, univariate Cox regression analysis indicated that pathologic T and risk score were DFS prognostic factors for CC patients (Figure 4A). Moreover, multivariate Cox regression analysis showed that the five‐mRNA signature was an independently DFS prognostic factor, after adjusting for stage T (Figure 4B). Then, the association between these factors and OS illustrated that the stage, pathologic T, pathologic N, pathologic M and risk score were OS prognostic factors for CC patients (Figure 4C). Subsequently, multivariate Cox regression analysis indicated that the five‐mRNA signature was an independently OS prognostic factor (Figure 4D). Taken together, above results showed that the risk score was an independently adverse prognostic factor for both DFS and OS of CC patients.

**FIGURE 5:**
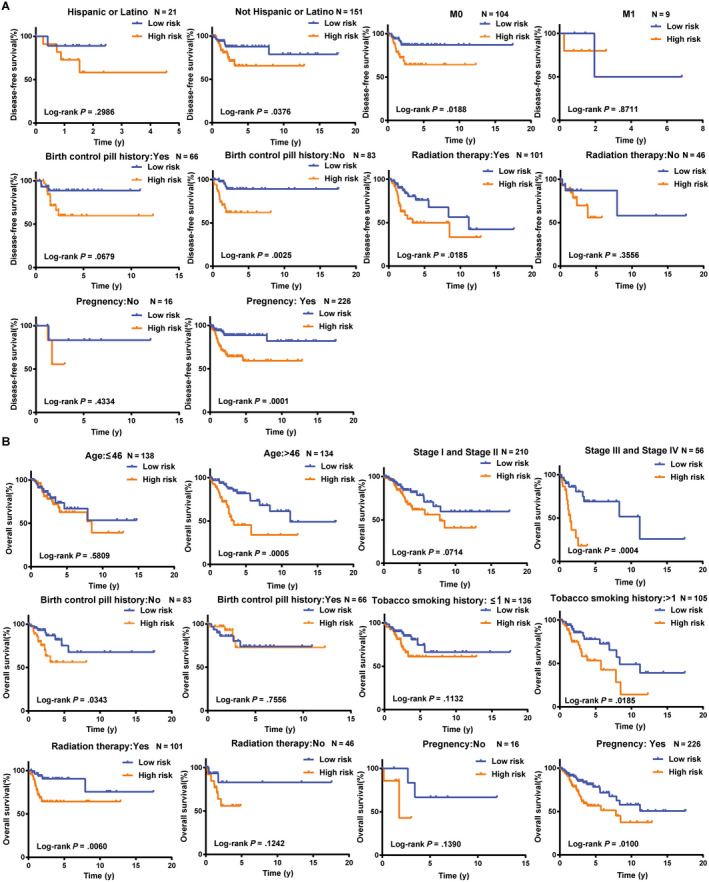
Kaplan‐Meier curves of DFS and OS for CC patients stratified by clinical features. A, K‐M curves for DFS. CC patients divided by ethnicity, pathologic M, birth control pill history, radiation therapy and pregnancy history, respectively. B, K‐M curves for OS. CC patients divided by age, stage, birth control pill history, tobacco smoking history, radiation therapy and pregnancy history, respectively. CC, cervical cancer; DFS, disease‐free survival; OS, overall survival

Furthermore, stratified analyses based on these clinical characteristics were carried out to identify the suitable patient groups of the risk score system. The results showed that the risk score remained the ability of predicting the DFS within each subgroup of age, stage and tobacco smoking history (Figure S1). However, the risk score remained an independent prognostic factor for the subgroup of not Hispanic or Latino, M0, no birth control pill history, receiving radiation therapy and experienced pregnancy (Figure 5A), implying that CC may be a disease that requires further explanation. As for OS, the risk score system was more suitable for subgroup of older than 46 years old, stage III + IV, no birth control pill history, tobacco smoking history more than 1 year, receiving radiation therapy and experienced pregnancy (Figure 5B). Moreover, the results of stratification analysis showed that high‐risk patients in each stratum of those clinical parameters had significantly shorter DFS and OS than low‐risk patients (*P* < .05).

### 3.5 Functional annotation of the downstream target genes of the five mRNAs in the signature

**FIGURE 6:**
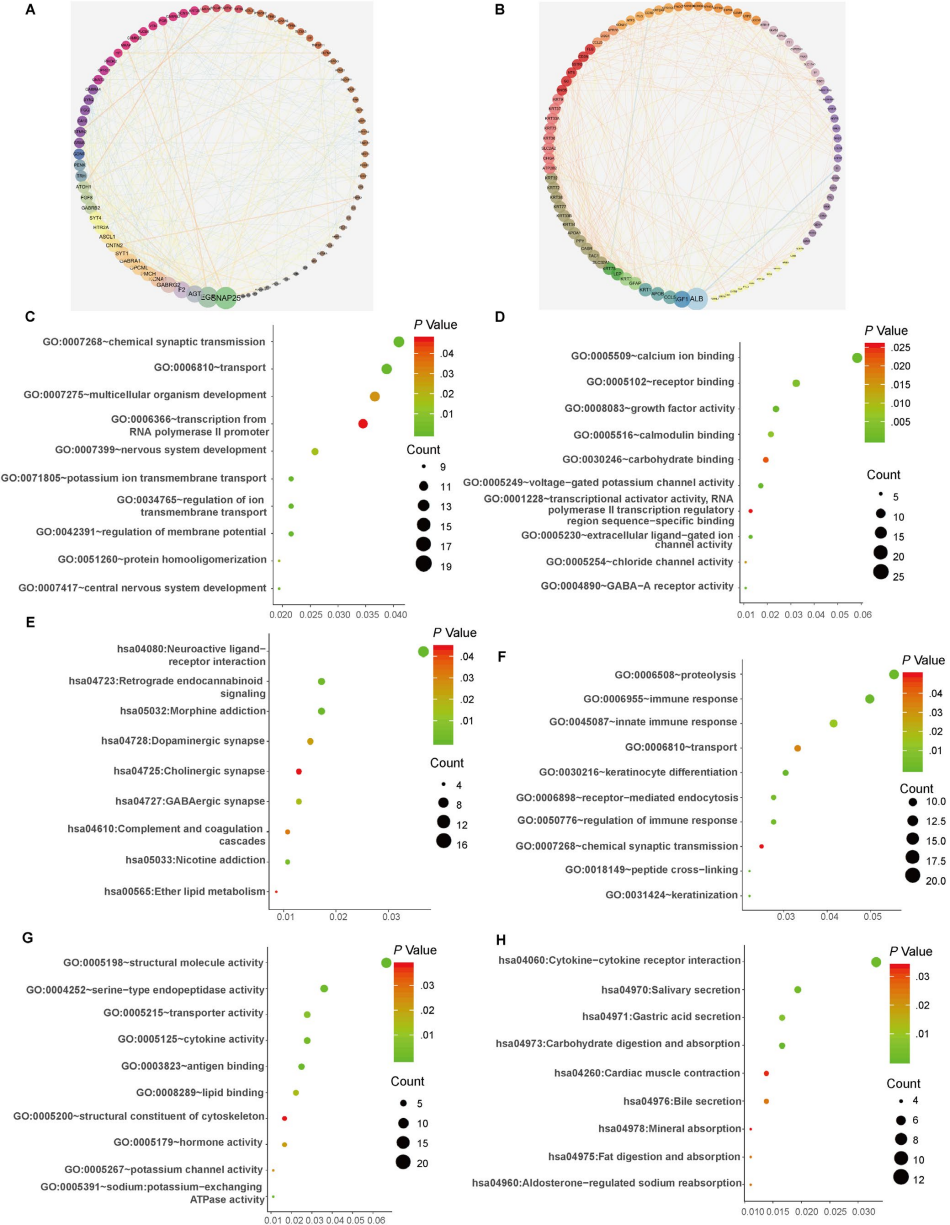
Functional enrichment analysis of the downstream target genes of the five mRNAs in the signature. A, Correction network of the up‐regulated DEGs visualized in Cytoscape. B, Correction network of the down‐regulated DEGs visualized in Cytoscape. The results of GO biological process enrichment (C), GO molecular function enrichment (D) and KEGG pathways analysis (E) of the up‐regulated DEGs, respectively. The results of GO biological process enrichment (F), GO molecular function enrichment (G) and KEGG pathway analysis (H) of the down‐regulated DEGs, respectively. DEGs, differentially expressed genes

The analysis of hub genes of up‐ and down‐regulated DEGs revealed that genes, such as SNAP25, EGF, AGT, F2 and GABRG2, could be seemed as the hub genes in up‐regulated DEGs (Figure 6A). On the other hand, such as ALB, IGF1, CCL5, APOB and KRT1, hub genes of down‐regulated DEGs were also found (Figure 6B). Subsequently, the results of functional annotation of the downstream target genes of the five mRNAs are shown in Figure 6 with top 10 most significant pathways in each functional access. And total enriched pathways are shown in Table S2. In up‐regulated DEG set, GO analysis results showed that genes were significantly enriched in ‘chemical synaptic transmission’, ‘receptor binding’ and ‘neuroactive ligand‐receptor interaction’ among biological processes (BP) (Figure 6C), molecular function (MF) (Figure 6D) and KEGG pathway(Figure 6E), respectively. Meanwhile, in down‐regulated DEG set, genes were significantly enriched in ‘immune response’, ‘antigen binding’ and ‘cytokine‐cytokine receptor interaction’ among BP (Figure 6F), MF (Figure 6G) and KEGG pathway (Figure 6H), respectively. In conclusion, the downstream target genes of the five mRNAs in the signature were associated with cell‐signalling networks in which PTM usually acted as significant regulatory switches. Furthermore, the down‐regulated DEGs were showed significant correlation with immunity.

### 3.6 Infiltrating immune cells as intrinsic features of CC patients can characterize the individual differences

A mass of researches has proved that PTMs usually affected many physiological processes, including immune function.^15^ Moreover, our previous results in this work also showed that the downstream target genes of the five mRNAs in the signature were associated with immunity in addition to cell‐signalling networks. Therefore, one emerging question is whether the PTM‐related signature can serve as a prognostic biomarker due to immunity. Then, we performed further analysis using CIBERSORT algorithm.

**FIGURE 7:**
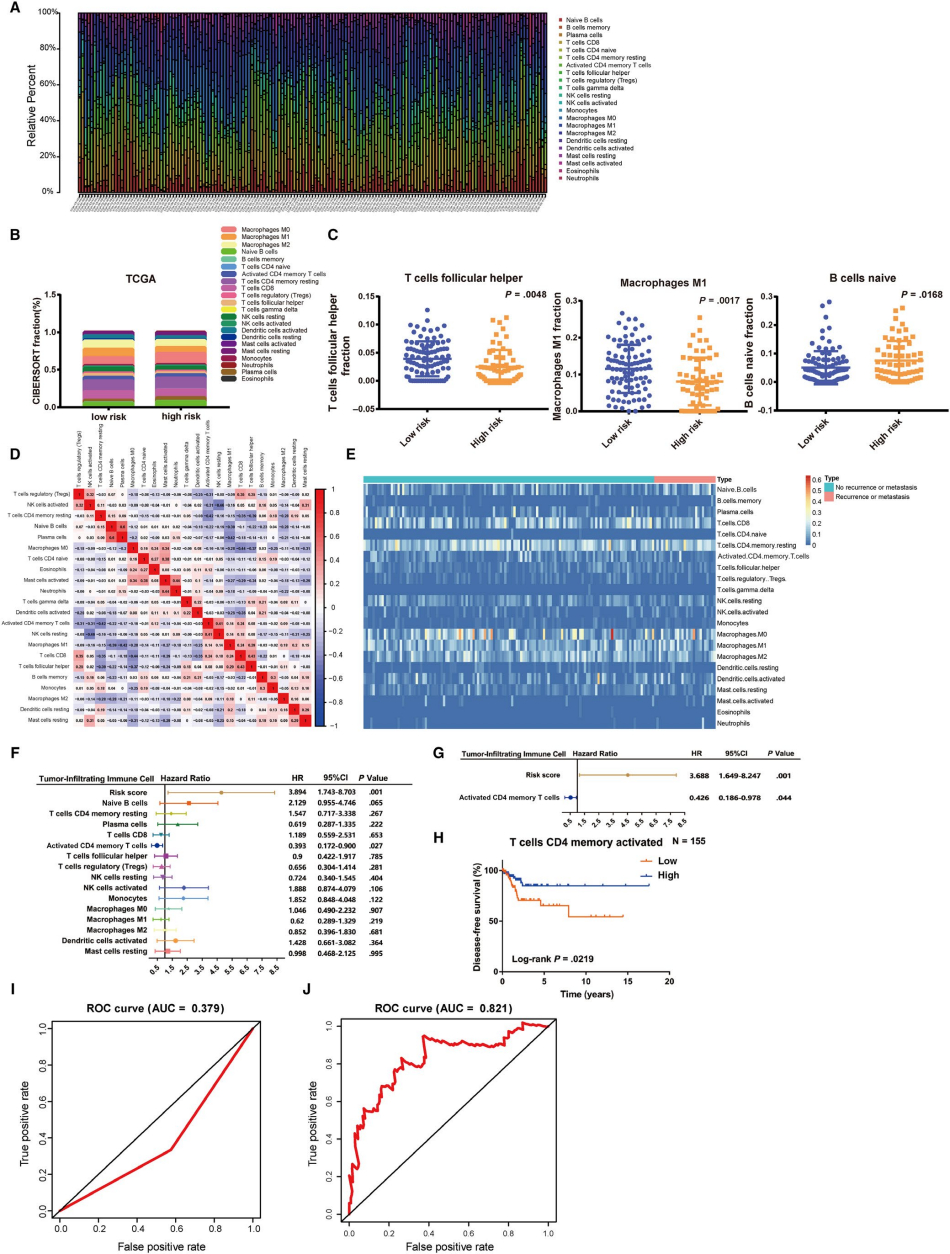
Infiltrating immune cells as intrinsic features of CC patients can characterize the individual differences and activated CD4 memory T cell is an independent factor for DFS. A, The summary of immune infiltration of 22 immune cells’ subpopulations in 155 samples. B, Composition of infiltrating immune cells in low‐risk and high‐risk groups. C, Different fractions of T cells follicular helper, macrophages M1 and naive B cell in low‐risk and high‐risk groups, respectively. D, Correlation matrix of all 22 immune cell proportions. E, Heatmap of the 22 immune cell proportions. F, Univariate Cox regression of prognostic associations of infiltrating immune cells for DFS. G, Multivariate Cox regression of prognostic associations of infiltrating immune cells for DFS. Unadjusted HRs (boxes) and 95% confidence intervals (horizontal lines) limited to cases with CIBERSORT *P*‐value < .05. Box size is inversely proportional to the width of the confidence interval. H, K‐M curves of activated CD4 memory T cell for DFS. I, ROC curve for DFS prediction by activated CD4 memory T cell. J, ROC curve for DFS prediction by the combination of activated CD4 memory T cell and five‐gene signature. CC, cervical cancer; DFS, disease‐free survival; HR, hazard ratio

Excluding samples which CIBERSORT *P*‐value > .05, 155 samples of the training set were enrolled. Figure 7A depicted a summary of immune infiltration of 22 subpopulations of immune cells in 155 samples. Detailed results are provided in Table S3. Significantly, the proportions of immune cells in CC varied obviously between each sample. We grouped 155 samples into low‐risk and high‐risk groups by cut‐off value of 0.902 as the median value of the whole training set risk score. And Figure 7B summarized the fraction of immune cells in the low‐risk and high‐risk groups, respectively. Then, we analysed difference in fraction of immune cells between low‐risk and high‐risk groups. The results showed that fraction of T cell follicular helper and Macrophages M1 was significantly higher in low‐risk groups than that in high‐risk groups (Figure 7C). However, fraction of naive B cell was remarkably higher in high‐risk groups than that in low‐risk groups. According to the result of Figure 7D, the proportions of different infiltrating immune cells subpopulations were weakly to moderately correlated. Unsupervised hierarchical clustering based on these immune cells was displayed in Figure 7E.

### 3.7 Activated CD4 memory T cell is also an independent factor for DFS of CC

Given above results, we can make a bold assumption that the PTM‐related signature can serve as a prognostic biomarker due to immunity. Thus, in order to verify our hypothesis, Cox regression was employed for analysis. Firstly, 7 subpopulations of immune cells were removed, because the number of samples with non‐expression exceeded half of the total number of samples. Then, the filtering resulted in 15 immune cell subpopulations that were used for further analysis. As results of univariate Cox regression analysis showed in Figure 7F, activated CD4 memory T cell (hazard ratio [HR] = 0.393, 95% CI = 0.172‐0.900; *P* = .027) was significantly associated with improved outcome. And the risk score (HR = 3.894, 95% CI = 1.743‐8.703; *P* = .001; Figure 7F) was DFS prognostic factors for CC patients consistent with our previous finding. Subsequently, multivariate Cox regression analysis results indicated that activated CD4 memory T cell and risk score were independent prognostic factors for DFS (HR = 0.426, 95% CI = 0.186‐0.978; *P* = .044; Figure 7G), which means those activated CD4 memory T cells could add additional prognostic value to our five‐mRNA signature for predicting DFS of CC patients.

Subsequently, K‐M curves showed that activated CD4 memory T cell was a prognostic biomarker for CC (*P* = .0219; Figure 7H). According to the curves, patients with low fraction of activated CD4 memory T cell had poorer prognoses. This observation further confirmed the accuracy of our analysis. Subsequently, we used ROC curves to investigate the predictive accuracy of activated CD4 memory T cell. The results revealed that activated CD4 memory T cell had little value for predicting DFS (AUC of 0.379; Figure 7I). Moreover, we combined the five‐gene risk score and the level of activated CD4 memory T cell. Surprisingly, the combination provided the best prediction performance for DFS (AUC of 0.821; Figure 7J), which means that activated CD4 memory T cell could add additional prognostic value to our PTM‐related signature for predicting DFS of CC patients.

## 4 DISCUSSION

A body of studies dedicated to discover prognostic biomarkers for CC. Although many prognostic indicators were already found in previous study,^16^, ^17^, ^18^, ^19^ only a limited number of gene‐combined prognostic signatures for CC were excavated out. And gene signatures that can predict both OS and DFS of CC have not yet been examined.

Post‐translational modifications were reported in numerous studies as its vital roles in life. However, when some genes, such as PTEN which was a major tumour suppressor, modified by PTMs, tumorigenesis could usually be accelerated in many cancers, such as CC.^20^ In contrast, if a normal procession of PTMs was inhibited, it will induce the promotion of cancer progression, such as CC.^21^ Given these, we recognized that PTMs had considerable links with tumorigenesis and metastasis in CC. Nevertheless, PTM‐related prognostic signatures for CC have not yet been examined.

Therefore, we mined five PTM‐related genes (GALNTL6, ARSE, DPAGT1, GANAB and FURIN) exhibited a significant prognostic value for DFS in CC patients. K‐M curves (log‐rank *P* < .0001) and ROC curves (AUC of 0.792) also indicated that these genes had a great value for predicting the 5‐year prognosis for CC. Surprisingly, we found that the five‐mRNA signature can also predict OS of CC patients(log‐rank *P* = .0030). And ROC curve indicated a high OS prediction performance (AUC of 0.705). In addition, the robustness of the signature was validated using GSE44001 (log‐rank *P* = .028). Analysis using HPA database proved that the expression of most of the five mRNAs was higher in CC tissues compared with normal tissues. Then, Cox regression analysis indicated that the five‐mRNA signature was an independently prognostic factor in CC patient.

In this study, we developed a prognostic five‐mRNA signature (GALNTL6, ARSE, DPAGT1, GANAB and FURIN) for CC. According to previous study, the expression of GALNTL6 was most frequently amplified in genome of papillary thyroid carcinomas.^22^ DPAGT1 has been reported that it had important impact on the procession of protein glycosylation which is one type of PTMs.^23^ In several studies, DPAGT1 was reported that it was associated with the progression of cancer^24^, ^25^ and could serve as a target for cancer treatment.^26^, ^27^ High expression of GANAB was significantly correlated with poor prognosis in melanoma.^28^ Moreover, Qin, Y et al illustrated that GANAB could act as an oncogene in gastric cancer.^29^ As for FURIN, previous study revealed that it was correlated with invasion and metastasis in CC.^30^ This discovery was consistent with our finding that FURIN was a negative indicator for CC. Furthermore, it was reported that FURIN could predict survival in ovarian cancer^31^ and might be seemed as a prognostic marker and therapeutic target for cancer.^32^, ^33^, ^34^ These studies might provide further evidence for our findings that the five genes were associated with poor prognosis.

However, among these five genes, GALNTL6, ARSE, DPAGT1 and GANAB have not been proved to be well characterized in CC except for FURIN. Therefore, we performed further analysis to better explore the clinical significance of these four genes in CC. Using GEPIA 2, we demonstrated that each of these four genes was significantly associated with DFS of CC and high expression of these four genes was associated with poor prognosis (Figure S2). Stratified analyses based on clinical characteristics revealed that all of the four genes’ expression remained independently prognostic factors for some subgroups of CC patients (Figure S3). Moreover, the results of these analyses showed that high‐expression patients in each stratum of those clinical parameters had significantly shorter DFS and OS than low‐expression patients. Above all, we have sufficient evidence to believe that the association between the genes and CC was strong. Collectively, these studies further confirmed our findings that the five‐mRNA signature could serve as a prognostic biomarker and was associated with poor prognosis of CC.

Many researches have mined lots of gene‐combined prognostic signature for CC. However, the five‐mRNA signature, dug in this study, showed better sensitivity and specificity of prediction for CC compared with other gene‐combined signatures reported in previous studies. For example, the AUC value of DFS of our signature is 0.792, and the value of reported signature is 0.747.^35^ As for OS, the AUC value is 0.705 in our study compared with the other value of 0.607 in reported research.^36^ Moreover, there is no gene‐combined signature reported can predict both DFS and OS of CC. Therefore, we could infer that our five‐mRNA signature might better predict both DFS and OS of CC. However, there were no experimental data to verify this conclusion. More clinical samples should be utilized to verify the predictive efficiency of the five‐mRNA signature in future studies.

Furthermore, functional annotations revealed that the downstream target genes of the five mRNAs were associated with cell‐signalling networks in which PTM usually acted as significant regulatory switches. The breakdowns of this signalling underlie many occurrence of disease, such as cancer and disorders of the immune systems.^37^ In addition, the down‐regulated DEGs were showed a significant correlation with immunity. These results hint us that the PTM‐related signature may serve as a prognostic biomarker for CC due to immunity.

Therefore, in order to verify this hypothesis, we further evaluated the infiltrating immune cells of CC using CIBERSORT algorithm. The proportions of immune cells in CC varied obviously between each sample. Then, we found that fraction of T cell follicular helper and macrophages M1 was significantly higher in low‐risk groups than those in high‐risk groups. However, fraction of naive B cell was remarkably higher in high‐risk groups, possibly indicating an impeded infiltration into tumour. These results illustrated that infiltrating immune cells as intrinsic features of CC patients could characterize the individual differences and might have clinical significance. Additionally, previous researches unveiled that PTMs usually showed crucial influence on the immune system, such as innate immunity and inflammatory responses.^38^ Meng et al proved that FBXO38, a specific E3 ubiquitin ligase of PD‐1, could degrade PD‐1 expression through Lys48‐linked poly‐ubiquitination in activated T cells.^39^ Cox regression analysis indicated that activated CD4 memory T cell was excavated as an independent prognostic factor for DFS, in addition to the risk score, and was associated with improved prognosis. Then, K‐M curves verified the above conclusions (*P* = .0219), and patients with low fraction of activated CD4 memory T cell had poorer prognosis. Previous study reported that memory T cell infiltration was commonly correlated with improved survival^40^, ^41^ and might control postoperative metastatic recurrence.^42^ These researches were consistent with our findings. ROC curves showed that the activated CD4 memory T cell had lower AUC value (0.379) than our five‐mRNA signature (0.792). Subsequently, the combination of activated CD4 memory T cell and the five‐mRNA signature showed high AUC value (0.821) which means that activated CD4 memory T cell could add additional prognostic value to our five‐mRNA signature for predicting DFS of CC patients. Above all, we can speculate that the five‐mRNA signature has better prediction performance than activated CD4 memory T cell and activated CD4 memory T cell may play an auxiliary role in predictive ability of our PTM‐associated signature and add additional prognostic value to it.

Despite the significant results obtained in the current study, there were inevitably several shortcomings of our study that should be acknowledged. First, gene expression data and associated clinical characteristics of CC patients were downloaded from publicly available data sets. However, the information of publicly available data sets is limited, so that the clinicopathological parameters analysed in this study are not comprehensive, which might induce bias results. Second, we did not consider the heterogeneity of the immune microenvironment related to the location of immune infiltration. Third, there were no experimental data regarding the identified signature. Therefore, further research is needed to elucidate the inherent correlation between the five‐mRNA signature and the prognosis of CC patients.

In conclusion, in this study, we mined an independent and robust five‐mRNA signature which could predict both DFS and OS for CC, and the signature showed better sensitivity and specificity of survival prediction compared with the results of some previous studies. Moreover, activated CD4 memory T cell was detected that it was an independent prognostic factor for DFS and could add additional prognostic value to our PTM‐associated signature. This is the pioneering research to identify a gene‐combined signature which can predict both OS and DFS of CC. In addition, the discovery also provides further insights for the prognostic predication of CC. Moreover, detailed analysis of the cellular immune response in CC has the potential to enhance clinical prediction and to identify candidates for immunotherapy. However, for clinic utilization and evaluating the prognostic value of our model, an integrative analysis combining the expression data and other variation data will be carried out in further studies.

## CONFLICT OF INTEREST

The authors declare that they have no conflict of interest.

## AUTHOR CONTRIBUTIONS

M Ju and A Qi: Conception, Integral analysis, Data mining, Writing‐original draft. M Wei and Lin Zhao: Conception, Writing‐review & editing. J Bi, Q Wei and L Jiang: Data downloading and processing. Lan Zhao, Q Zhang and X Li: GSEA analysis. Q Guan and L Wang: Data statistics.

## Supporting information

Fig S1‐S3

Table S1

Table S2

Table S3

## Data Availability

The original TCGA data that support the findings of our study are available in the NCI GDC Data portal repository at the following URL: https://portal.gdc.cancer.gov/repository. Gene Expression Omnibus (GEO, https://www.ncbi.nlm.nih.gov/geo/) data were from NCBI GEO (accession numbers: GSE14520).
