# Supplementary material for: A five‐mRNA signature associated with post‐translational modifications can better predict recurrence and survival in cervical cancer
Source: J Cell Mol Med. 2020 Apr 19;24(11):6283–97. doi: 10.1111/jcmm.15270 (PMC7294153; doi:10.1111/jcmm.15270)
Supplement: Supplementary file 1 — Fig S1‐S3 [file JCMM-24-6283-s001.docx]

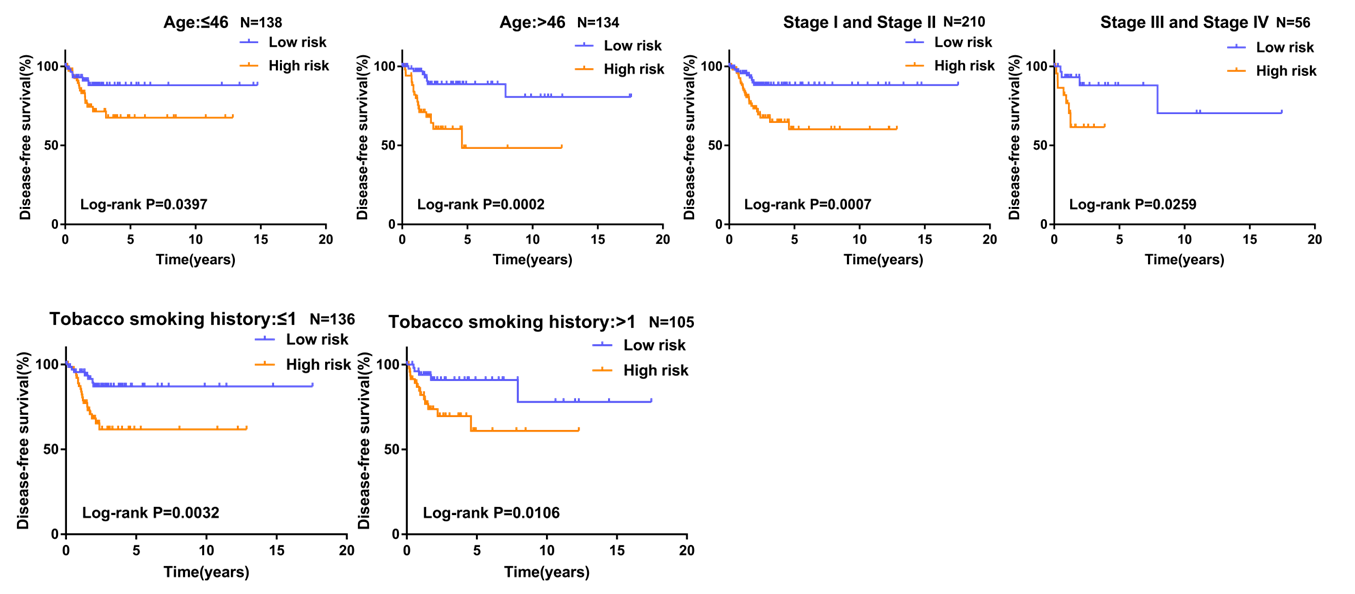


Figure S1. K-M curves for DFS. CC patients divided by age, stage and tobacco smoking history, respectively.


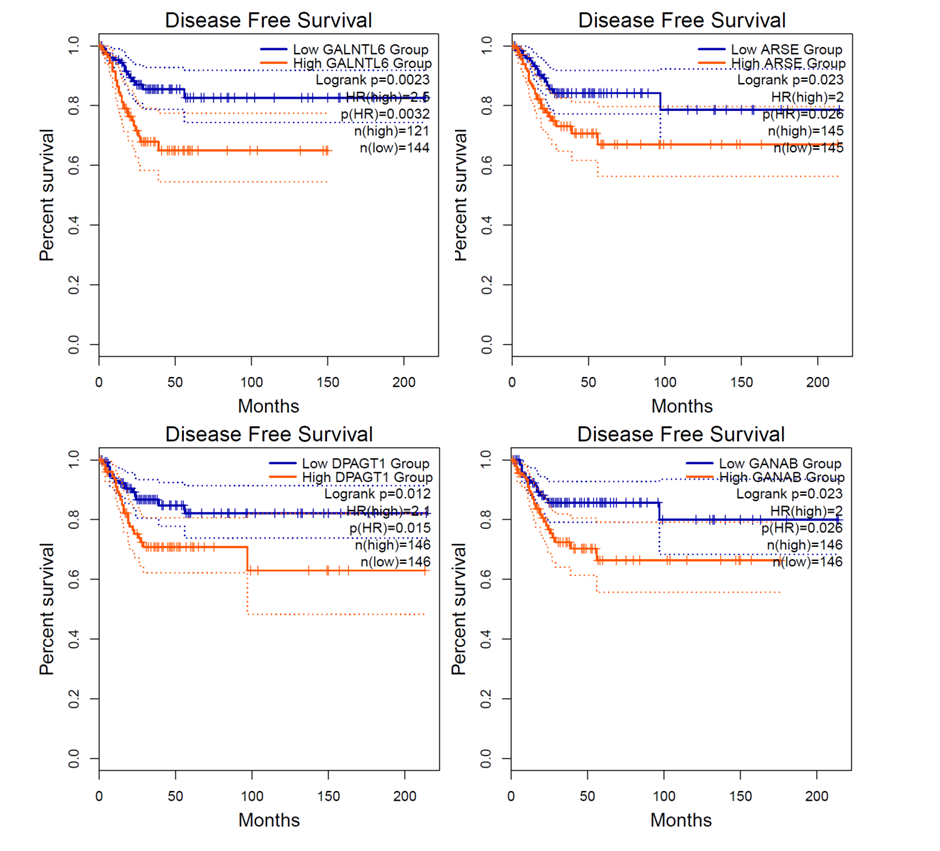


Figure S2. K-M curves for DFS by the expression of GALNTL6, ARSE, DPAGT1 and GANAB, respectively.


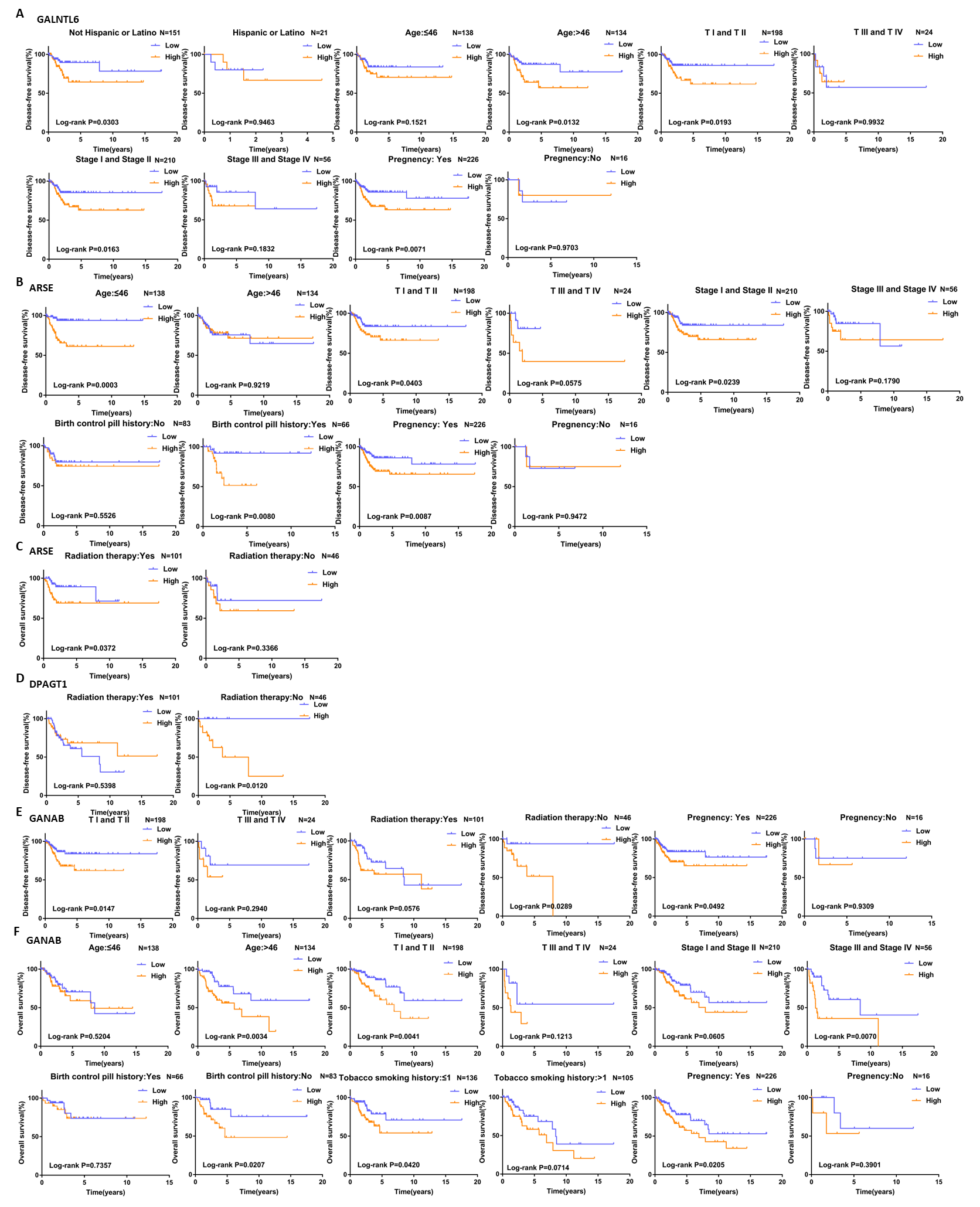


Figure S3. K-M curves of DFS and OS for CC patients stratified by clinical features. K-M curves for DFS (A) by the expression of GALNTL6. K-M curves for DFS (B) and OS (C) by the expression of ARSE. K-M curves for DFS (D) by the expression of DPAGT1. K-M curves for DFS (E) and OS (F) by the expression of GANAB.
